# Beyond re-epithelialization: prolonged remodeling re-establishes epithelial homeostasis in oral mucosa

**DOI:** 10.1038/s41419-026-08804-z

**Published:** 2026-04-28

**Authors:** Carson Joseph Walton, Tyler Thompson, Sofia Ali Syed, Tianli Zhu, Ella Guo, Xue Yuan

**Affiliations:** 1 Indiana University School of Medicine, Department of Otolaryngology-Head & Neck Surgery, Indianapolis, IN USA; 2 Indiana University School of Medicine – South Bend, South Bend, IN USA; 3 Indiana University School of Dentistry, Department of Biomedical Sciences and Comprehensive Care, Indianapolis, IN USA; 4 Carmel High School, Carmel, IN USA; 5 Indiana University School of Medicine, Indiana Center for Musculoskeletal Health, Indianapolis, IN USA; 6 Indiana University Simon Comprehensive Cancer Center, Indiana University School of Medicine, Indianapolis, IN USA

**Keywords:** Apoptosis, Regeneration

## Abstract

Wound healing restores tissue integrity through tightly coordinated cellular and molecular programs, yet the biological processes that persist after apparent closure remain largely unexplored. Using a standardized mouse hard palate injury model combined with histological, lineage-tracing, molecular, and epigenetic analyses, we show that rapid re-epithelialization is followed by months of cellular remodeling. Although wounds re-epithelialized within two weeks, temporal analyses revealed that the regenerated epithelium maintained leading-edge-like features for months. Structural reorganization, including increased cell density and epithelial thickness, persisted well beyond closure, accompanied by sustained proliferation and elevated apoptosis mediated by the p53-p21 pathway, thereby removing cells with DNA damage during tissue remodeling. Lineage tracing revealed marked alterations in Wnt-responsive epithelial stem cells, which were transiently lost from the repaired epithelium and gradually repopulated the tissue over approximately four months. Repair-activated keratinocytes showed sustained expression of keratin 6 and keratin 17 and delayed recovery of differentiation markers keratin 10 and filaggrin, indicating prolonged epithelial activation despite morphological closure. Coordinated alterations in histone marks H2AK119ub, H3K27ac, and H3K27me3, and persistent immune infiltration, indicate lasting molecular and microenvironmental remodeling that maintains the tissue in a memory-like state. Together, these findings indicate that oral wound healing is an extended, multi-phase process in which post-healing remodeling restores epithelial homeostasis and ensures proper resolution of the repair response.

## Introduction

Wound healing is a fundamental biological process that restores tissue integrity after injury through tightly regulated cellular and molecular programs [1, 2]. It involves dynamic coordination among multiple cell types and signaling pathways across four overlapping phases—hemostasis, inflammation, proliferation, and remodeling—that collectively reestablish barrier function and ensure structural and functional recovery [3, 4]. Although the early phases have been extensively characterized, the remodeling phase and the mechanisms governing the reestablishment of tissue homeostasis remain insufficiently defined. Defining this phase is critical for understanding how adult tissues orchestrate the activation and resolution of regenerative programs, and how their dysregulation underlies chronic wounds, fibrosis, and malignancy.

Epithelial stem cells play a central role in tissue repair by replenishing lost cells and restoring epithelial integrity [5–8]. Following injury, they are rapidly activated from quiescence, undergoing proliferation and transient transcriptional plasticity to generate progenitors that migrate, divide, and differentiate to close the wound [9, 10]. Live imaging and lineage-tracing studies have revealed that wound closure involves a spatiotemporal coordination of migration and proliferation, where transient progenitors drive rapid epithelial expansion during repair, and long-lived stem cells subsequently reestablish the self-renewing pool that supports long-term tissue maintenance [11, 12]. However, these studies have primarily focused on the re-epithelialization phase, which ends once the wound site is covered. How epithelial stem cells contribute to the subsequent remodeling phase and how the tissue gradually regains its homeostatic organization remain largely unknown.

Recent studies of skin repair have revealed that epithelium can retain molecular “memories” that influence subsequent regenerative responses [13, 14]. Epithelial stem cells can undergo epigenetic “pre-activation” driven by proinflammatory cytokines and morphogen-like growth factors, which prime them for accelerated repair upon re-injury [15, 16]. While this adaptive memory enhances regenerative efficiency, it may also confer a persistent, plastic state that promotes aberrant proliferation or field cancerization, linking the post-wound microenvironment to long-term cancer susceptibility [16–19]. Despite these insights, how epithelial stem cells in the oral mucosa transition from a transient repair-activated state to a stable homeostatic niche remains unknown. In contrast to skin, oral wounds heal rapidly with minimal scarring and inflammation, suggesting that remodeling may be governed by context-specific regulatory mechanisms [20, 21]. Understanding these mechanisms is essential for defining how epithelial tissues accomplish long-term regeneration without progressing toward pathological remodeling.

Here, we combined histological, lineage-tracing, molecular, and epigenetic analyses to systematically characterize oral epithelial remodeling up to six months after injury. We show that oral wound healing proceeds in two phases: rapid re-epithelialization is followed by a prolonged remodeling stage marked by removal of repair-activated cells, delayed reorganization of stem cells, sustained stress-keratin expression, and persistent immune infiltration. Mechanistically, removal of repair-activated cells is associated with DNA damage-activated p53-p21 signaling, which leads to cell-cycle arrest and apoptosis, accompanied by coordinated epigenetic remodeling. Together, these findings redefine oral wound healing as an extended, multi-phase process in which post-healing remodeling serves as a critical checkpoint that restores epithelial integrity and prevents maladaptive repair.

## Results

### Dynamics of epithelial structure and cellular activities during post-healing remodeling

To investigate post-healing dynamics, we employed a mouse hard palate injury model with 2-mm circular wounds created between the first molars. The palatal rugae provided reliable anatomical landmarks, ensuring consistent orientation and reproducibility (Supplementary Fig. 1A, B). Wound closure was consistent and completed by day 14, and the fixed positions of rugae and molars enabled precise localization of the healed sites for downstream analyses (Supplementary Fig. 1C-E).

**Fig. 1:**
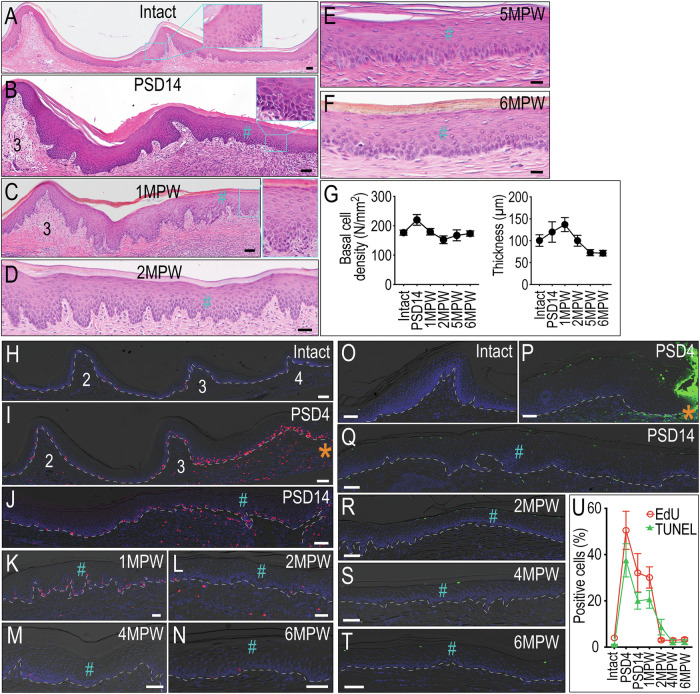
Histological and cellular dynamics of the epithelium during post-healing remodeling. Representative H&E-stained sections of the oral epithelium at different time points: **A** intact, **B** post-surgery day 14 (PSD14), **C** 1 month post-wound (1MPW), **D** 2MPW, **E** 5MPW, and **F** 6MPW. Insets show higher-magnification views of boxed regions. **G** Quantification of basal cell density (*n* = 8) and epithelial thickness (*n* = 3) at the indicated time points. **H**–**N** Representative EdU staining showing proliferating epithelial cells. **O**–**T** Representative TUNEL staining showing apoptotic epithelial cells. **U** Quantification of EdU^+^ and TUNEL^+^ cells (% of total epithelial cells) across time points (*n* = 8). Data are presented as mean ± SD. Dashed lines outline the epithelial-dermal boundary. The healed area is marked by # symbols. Numbers 2, 3, and 4 indicate hard palate rugae 2–4, respectively. Asterisks (*) denote wound sites. Scale bars, 50 μm.

At PSD14, immediately post-closure, the epithelium exhibited markedly increased cellularity relative to intact tissue (Fig. 1A, B, quantified in G). The basal layer showed increased cell density, with basal keratinocytes appearing more crowded and compressed compared to the typical columnar morphology observed in intact tissue. By one month post-wound (1MPW), density declined toward baseline and morphology was restored (Fig. 1C, quantified in G). At 2MPW, basal cell density further declined below that of the intact epithelium (Fig. 1D, quantified in G). Cell density gradually recovered at 5MPW and reached near-baseline levels by 6MPW (Fig. 1E, F, quantified in G). Meanwhile, epithelial thickness increased after injury, peaking at 1MPW, then returned to baseline by 2MPW and decreased below baseline at later stages (Fig. 1C-F, quantified in G).

To further elucidate the cellular dynamics underlying epithelial remodeling, we analyzed proliferation using EdU labeling to mark cells undergoing DNA synthesis. Following injury, proliferation surged sharply, peaking at PSD4 (Fig. 1H, I, quantified in U). Although the wound had closed by PSD14, EdU-positive cells remained abundant, and proliferation stayed above baseline through 1MPW (Fig. 1J, K, quantified in U). Thereafter, proliferating cells declined rapidly, returning to near-intact levels by 2MPW and remaining low afterward (Fig. 1L-N, quantified in U). Given the sustained proliferative activity beyond wound closure, apoptosis was examined as a compensatory mechanism to restore balance and prevent cell accumulation. TUNEL staining revealed a few apoptotic cells in intact epithelium (Fig. 1O). After injury, apoptosis increased sharply and remained high through PSD14 (Fig. 1P, Q, quantified in U). At 2MPW, apoptotic activity persisted above baseline (Fig. 1R, quantified in U), then declined by 4MPW and returned to normal at 6MPW (Fig. 1S, T, quantified in U). Co-immunostaining with Keratin 6 and CD45 at PSD14 confirmed that TUNEL-positive cells were predominantly epithelial rather than immune in origin (Supplementary Fig. 2).

These data indicate that injury triggers a robust epithelial expansion that extends beyond wound closure, followed by a prolonged apoptotic phase that restores homeostasis.

### Molecular mechanisms underlying persistent epithelial cell apoptosis following wound healing

**Fig. 2:**
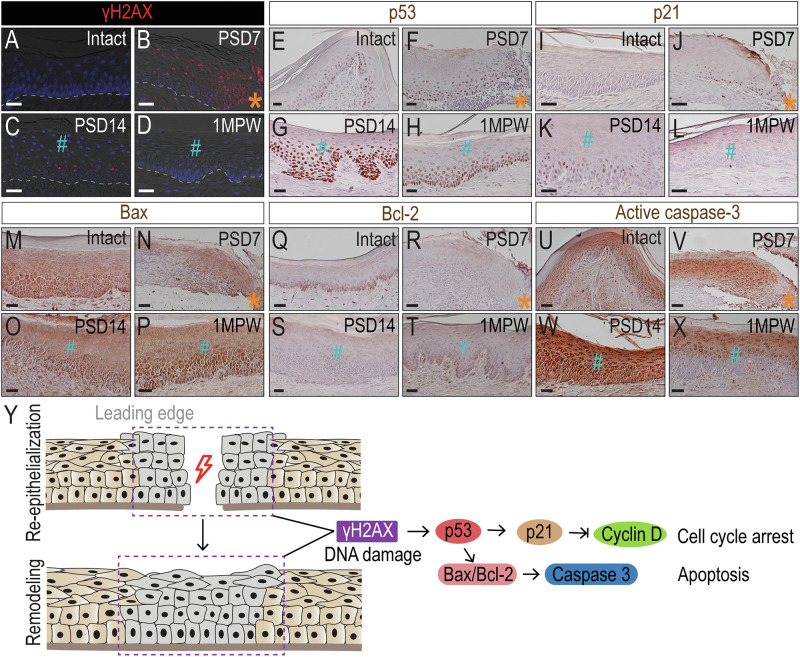
Molecular mechanisms underlying persistent epithelial cell death following wound healing. **A**–**D** Representative immunofluorescence images showing γH2AX expression (red) at the indicated time points. Representative immunohistochemical staining for (**E**–**H**) p53, (**I**–**L**) p21, (**M**–**P**) pro-apoptotic Bax, (**Q**–**T**) anti-apoptotic Bcl-2, and (**U**–**X**) active caspase-3. **Y** Schematic model illustrating the DNA damage-p53-p21 signaling cascade and its downstream effects on cell-cycle arrest (via Cyclin D1) and apoptosis (via Bax/Bcl-2-Caspase 3) during post-wound remodeling. Dashed lines outline the epithelial-stromal boundary. The healed area is indicated by # symbols, and asterisks (*) denote wound sites. Scale bars, 25 μm.

To elucidate the molecular basis of persistent apoptosis after re-epithelialization, we first assessed DNA damage dynamics by γH2AX staining. γH2AX-positive cells were abundant at the wound edge during early repair (Fig. 2A, B). Notably, a considerable number of DNA-damaged cells persisted within the newly re-epithelialized tissue at PSD14 (Fig. 2C), suggesting incomplete resolution of DNA lesions. By 1MPW, γH2AX-positive cells had largely disappeared, indicating effective repair or clearance of damaged cells (Fig. 2D). We next examined p53, a key effector of the DNA damage response, and found markedly elevated levels in regions showing strong γH2AX signals, consistent with its rapid activation following DNA insult (Fig. 2E-H).

Downstream of p53, we examined its target p21, a cyclin-dependent kinase inhibitor essential for p53-mediated cell cycle arrest, and found it strongly induced at PSD7 with strong localization at the leading edge of the regenerating epithelium (Fig. 2I, J). p21-positive cells were evident within the healed site (Fig. 2K), overlapping regions of γH2AX and p53 activation. At 1MPW, p21 levels declined but remained detectable (Fig. 2L). Consistent with p21 induction, Cyclin D1 and PCNA levels were reduced in the remodeling epithelium (Supplementary Fig. 3A-H). Simultaneously, analysis of the Bcl-2 family revealed dynamic changes in Bax/Bcl-2 ratio, shifting in favor of Bax (Fig. 2M-T), which is pro-apoptotic. We also observed elevated active caspase-3 (Fig. 2U-X), indicating apoptotic activation in the re-epithelialized tissue.

Quantitative PCR analysis confirmed these findings at the transcriptional level. At PSD14, Trp53 and its target genes (*Cdkn1a*, *Mdm2*, *Pmaip1*, *Gadd45a*) showed robust upregulation, validating sustained p53 pathway activation in healed epithelium (Supplementary Fig. 3I). Expression of the anti-apoptotic gene Bcl2 decreased significantly, while Bax remained unchanged (Supplementary Fig. 3I), consistent with the pro-apoptotic shift observed at the protein level. By 1MPW, target gene expression declined but remained above baseline (Supplementary Fig. 3I), paralleling the protein dynamics and confirming prolonged p53-mediated remodeling after wound closure.

Taken together, these data delineate a cascade in which DNA damage triggers γH2AX activation, leading to p53 induction. Activated p53 upregulates p21 to enforce cell-cycle arrest through Cyclin D1 inhibition and concurrently shifts the Bax/Bcl-2 balance toward Bax, activating caspase-3 for apoptosis (Fig. 2Y). This cascade provides a mechanistic basis for the sustained epithelial cell death during remodeling, enabling clearance of damaged cells and reestablishment of homeostasis after wound closure.

### Limited incorporation of Axin2^+^ Wnt-responsive stem cells in early regenerated oral epithelium

We previously demonstrated that Axin2^+^ Wnt-responsive cells serve as epithelial stem cells in the oral epithelium [7, 22, 23]. Given the continued remodeling of healed tissue, we sought to determine when Wnt-responsive cells reestablish homeostasis within the regenerated epithelium.

**Fig. 3:**
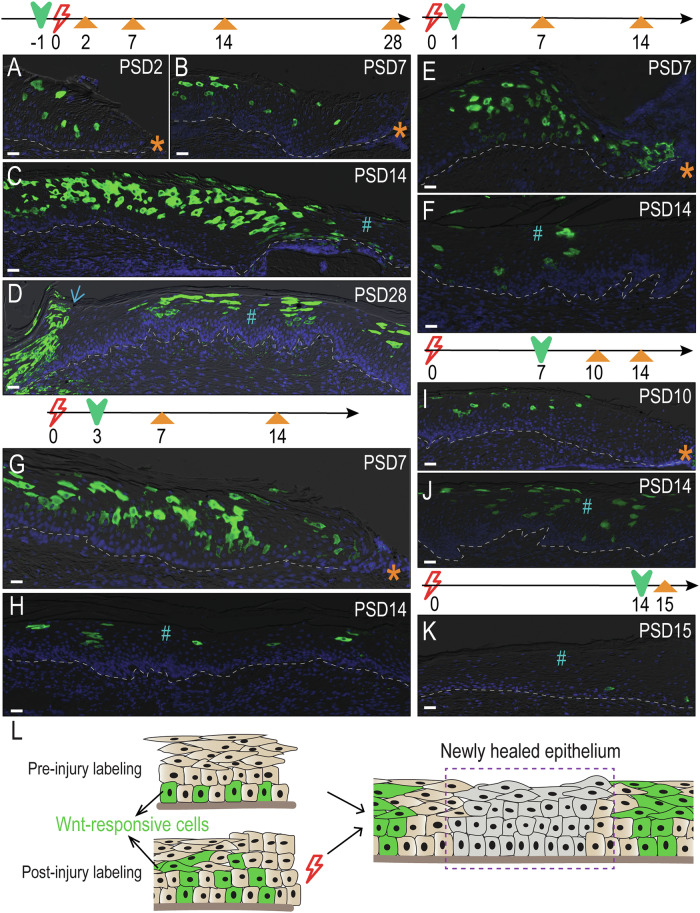
Limited incorporation of Axin2^+^ Wnt-responsive stem cells during early oral epithelial regeneration. **A**–**D** *Axin2*^*CreERT2*^*;EGFP* mice were administered tamoxifen one day prior to injury (day -1) to permanently label Axin2^+^ Wnt-responsive cells. Representative immunofluorescence images show EGFP^+^ cell distribution at the indicated time points. **E**, **F** Mice administered tamoxifen at post-surgery day 1 (PSD1) and analyzed at PSD7 and PSD14. **G**, **H** Mice administered tamoxifen at PSD3 and analyzed at PSD7 and PSD14. **I**, **J** Mice administered tamoxifen at PSD7 and analyzed at PSD10 and PSD14. **K** Mice administered tamoxifen at PSD14 and analyzed at PSD15. **L** Schematic of lineage tracing of Axin2⁺ Wnt-responsive cells. Pre- and post-injury labeling strategies are shown, illustrating their limited contribution to the newly healed epithelium. Timeline diagrams above images indicate tamoxifen injection (green arrowhead), injury (red lightning bolt), and analysis (orange triangles) time points. Dashed lines outline the epithelial–dermal boundary. The healed area is indicated by # symbols. Asterisks (*) denote wound sites. Scale bars, 50 μm.

In *Axin2*^*CreERT2*^*;EGFP* lineage-tracing mice, tamoxifen was administered one day before injury to label Axin2⁺ cells with EGFP expression. During re-epithelialization, EGFP⁺ cells were scattered along wound edges but were absent from the basal layer within the epithelial front (Fig. 3A, B). Interestingly, by PSD14, EGFP⁺ cells were largely absent from the newly formed epithelium covering the healed wound, while those in distal tissue appeared to exit the basal layer and migrate toward the healed region (Fig. 3C). At PSD28, EGFP⁺ cells reappeared within the healed epithelium, displaying a distinct distribution pattern compared with adjacent uninjured areas and delineating a clear boundary between them (Fig. 3D, blue arrow).

To assess whether injury-induced Wnt-responsive cells contribute to the regenerated tissue, we performed tamoxifen labeling at different post-injury intervals. Labeling at PSD1 resulted in abundant EGFP⁺ cells at the leading edge by PSD7 (Fig. 3E) but minimal incorporation into the healed epithelium (Fig. 3F). Similarly, labeling at PSD3 demonstrated active EGFP⁺ cell involvement at PSD7 (Fig. 3G) but only sparse integration at PSD14 (Fig. 3H). Labeling at PSD7 showed a comparable trend, with EGFP⁺ cells detectable at PSD10 but largely absent by PSD14 (Fig. 3I, J). Lastly, labeling at PSD14 revealed few Wnt-responsive cells within the regenerated epithelium (Fig. 3K).

Collectively, these results indicate that Wnt-responsive cells, whether homeostatic or injury-induced, show limited incorporation into the newly regenerated oral epithelium (Fig. 3L). This pattern aligns with our apoptosis data, suggesting that many leading-edge cells are eliminated during remodeling despite rapid wound closure.

### Delayed re-establishment of Wnt-responsive stem cells in healed oral epithelium following remodeling

**Fig. 4:**
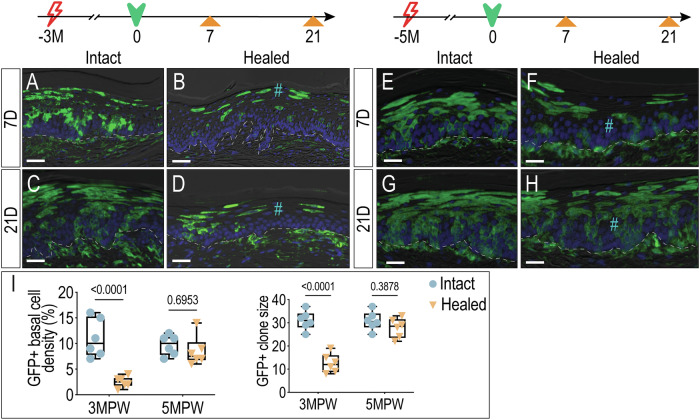
Delayed re-establishment of Axin2^+^ Wnt-responsive stem cells during post-healing remodeling. **A**–**D** *Axin2*^*CreERT2*^*;EGFP* mice were administered tamoxifen three months post-injury to label Axin2⁺ Wnt-responsive cells, and representative immunofluorescence images show EGFP⁺ cells at the indicated time points. **E**–**H** *Axin2*^*CreERT2*^*;EGFP* mice were administered tamoxifen five months post-injury, and representative immunofluorescence images show EGFP⁺ cells at the indicated time points. **I** Quantification of EGFP⁺ basal cell density and clone size comparing intact and healed tissue at 3MPW and 5MPW (*n* = 6). Data represent individual mice with mean ± SD. The healed area is marked by # symbols. Dashed lines outline the epithelial–dermal boundary. Scale bars, 50 μm.

To determine when the Wnt-responsive cell population is reestablished after healing, we performed lineage tracing at stages when proliferation and apoptosis had largely returned to baseline. In intact tissue, EGFP⁺ cells exhibited typical clonal architecture, forming coherent clones extending from basal to suprabasal layers (Fig. 4A, C), consistent with homeostatic Wnt-responsive cell organization as we reported previously [7]. In contrast, 3MPW healed tissue contained scattered EGFP⁺ cells that lacked basement membrane association and the characteristic basal-to-suprabasal clonal structure (Fig. 4B, D, quantified in I). When tamoxifen labeling was conducted at 5MPW, the healed epithelium closely resembled the intact counterpart, displaying a reestablished clonal organization from basal to suprabasal layers (Fig. 4E-H). Correspondingly, both EGFP⁺ basal cell density and clone size were comparable between intact and healed tissue at this stage, indicating full restoration (Fig. 4I). These results demonstrate that newly healed epithelium initially lacks a fully organized Wnt-responsive stem cell compartment but gradually regains homeostatic stem cells.

### Molecular and structural markers reveal prolonged epithelial activation after re-epithelialization

Lineage tracing revealed that Wnt-responsive cells were not fully restored until months after wound closure, prompting us to assess when the regenerated epithelium had regained normal differentiation and function. We analyzed key epithelial markers representing basal progenitor state (keratin 5), differentiation (keratin 10 and filaggrin), and wound-associated activation (keratin 6 and keratin 17), together with structural (E-cadherin) and basement membrane (Laminin 5) components.

**Fig. 5:**
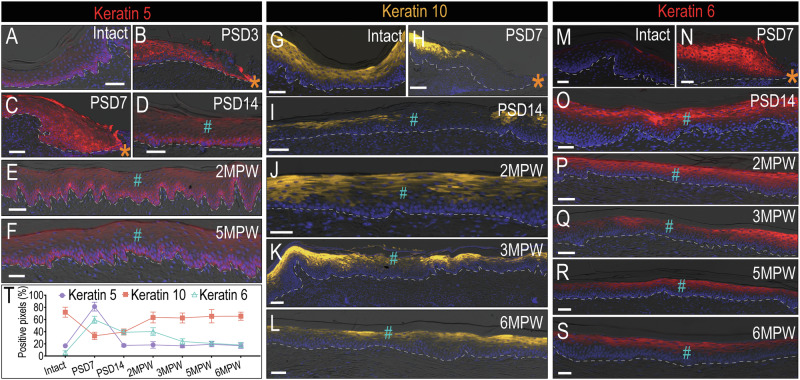
Keratin expression dynamics reflect prolonged epithelial activation during post-wound healing. Representative immunofluorescence staining of (**A**–**F**) keratin 5, (**G**–**L**) keratin 10, and (**M**–**S**) keratin 6 in the epithelium at the indicated time points. **T** Quantification of keratin 5, keratin 10, and keratin 6 expression intensity across all time points (*n* = 8). Data are presented as mean ± SD. Dashed lines outline the epithelial-stromal boundary. The healed area is marked by # symbols. Asterisks (*) denote wound sites. Scale bars, 50 μm.

Keratin 5 was confined to basal cells in intact tissue (Fig. 5A) but became strongly upregulated across all epithelial layers after injury (Fig. 5B, C). By PSD14, it returned to the basal layer, which was maintained through remodeling, though expression remained slightly elevated (Fig. 5D-F, quantified in T). Quantitative PCR analysis confirmed the dynamic changes in Krt5 expression at the transcriptional level (Supplementary Fig. 4A). Keratin 10 was abundant in suprabasal layers of intact tissue (Fig. 5G) but was reduced at the wound edge and remained low at PSD14 (Fig. 5H, I), indicating a differentiation defect. Keratin 10 expression gradually recovered, approaching the intact pattern by late stages (Fig. 5J-L, quantified in T). Filaggrin, a terminal differentiation marker, followed a similar course—absent early, reappearing at 2MPW, and restored by 6MPW (Supplementary Fig. 4B-H). Consistent with the immunostaining results, quantitative PCR further validated the temporal recovery of Flg expression (Supplementary Fig. 4I).

Keratin 6 was nearly absent in intact epithelium (Fig. 5M) but was rapidly and strongly induced at the wound edge, remaining elevated through 2MPW (Fig. 5N-P) and declining gradually thereafter (Fig. 5Q-S). Keratin 17 showed a comparable pattern (Supplementary Fig. 4J-P). These findings indicate a prolonged stress-activated epithelial state persisting beyond re-epithelialization.

E-cadherin expression, marking adherens junctions, remained largely stable throughout healing (Supplementary Fig. 5A-G). Laminin 5, a key basement membrane component, was markedly reduced immediately after injury but gradually recovered, restoring a continuous basement membrane pattern after 3MPW (Supplementary Fig. 5H-N).

In addition to epithelial restoration, the underlying connective tissue also underwent remodeling. Type I collagen, a major extracellular matrix component, was disrupted early but fully re-established by 3MPW (Supplementary Fig. 6A-E), whereas Vimentin expression remained low in the healed region even at 6MPW (Supplementary Fig. 6F-I).

Together, these results indicate that epithelial and stromal restoration proceeds gradually during an extended remodeling phase.

### Dynamic and spatially restricted immune cell infiltration persists during long-term post-wound remodeling

**Fig. 6:**
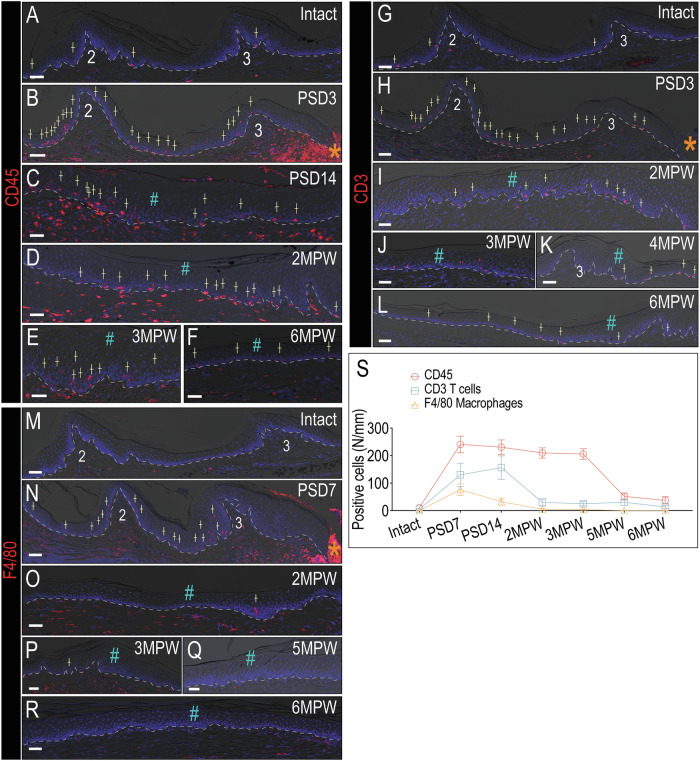
Temporal dynamics of immune cell infiltration and epithelial activation during post-wound oral mucosa remodeling. Representative immunofluorescence staining of (**A**–**F**) CD45^+^ leukocytes, (**G**–**L**) CD3^+^ T cells, and (**M**–**R**) F4/80^+^ macrophages. **S** Quantification of immune cell numbers within the epithelial layer (*n* = 8). Dashed lines outline the epithelial-stromal boundary. The healed area is marked by # symbols; † symbols denote positive immune cells within the epithelium. Numbers 2 and 3 indicate hard palate rugae landmarks. Asterisks (*) denote wound sites. Scale bars, 50 μm.

To further characterize the tissue microenvironment, we examined the temporal and spatial dynamics of immune cell populations during wound healing. CD45⁺ leukocytes markedly accumulated at wound sites after injury, including substantial intraepithelial infiltration (Fig. 6A-C). Their numbers declined after 2MPW and approached, but did not fully reach, baseline (Fig. 6D-F, quantified in S). Specifically, CD3⁺ T cells showed a marked increase during early healing (Fig. 6G, H). Although reduced by 2MPW, intraepithelial T cells remained above baseline through 6MPW (Fig. 6I-L, quantified in S). F4/80⁺ macrophages were rarely detected in intact epithelium (Fig. 6M) but increased substantially after injury (Fig. 6N). Their numbers gradually declined and returned to baseline by 5MPW (Fig. 6O-R, quantified in S).

MPO⁺ neutrophils accumulated in the stroma during acute injury but did not infiltrate the epithelium and rapidly declined during remodeling (Supplementary Fig. 7A-D). CD20^+^ B cells were limited to stromal regions and did not infiltrate the epithelium at any stage, with no noticeable increase following injury (Supplementary Fig. 7E-H).

These findings reveal that the epithelial immune landscape remains altered for months after healing.

### Epigenetic silencing underlies deficient protein recovery following wound healing

**Fig. 7:**
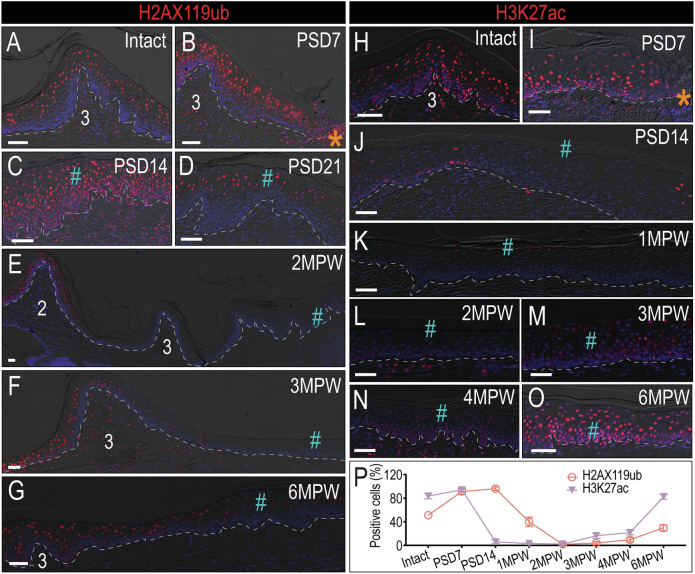
Temporal dynamics of histone modifications during wound healing and post-healing remodeling. Representative immunofluorescence staining of (**A**–**G**) H2AK119ub and (**H**–**O**) H3K27ac in the oral epithelium at the indicated stages of wound healing and remodeling. **P** Quantification of H2AK119ub^+^ and H3K27ac^+^ cells as a percentage of total epithelial cells (*n* = 8). Dashed lines outline the epithelial–stromal boundary. The healed area is marked by # symbols. Numbers 2 and 3 indicate hard palate rugae landmarks. Asterisks (*) denote wound sites. Scale bars, 50 μm.

As epithelial and immune recovery extended beyond re-epithelialization, we next examined whether epigenetic regulation underlies these sustained changes. We first analyzed histone H2A lysine 119 monoubiquitination (H2AK119ub), a Polycomb repressive complex 1 (PRC1)-associated repressive mark involved in epithelial memory [15]. In intact, H2AK119ub was detected predominantly in the suprabasal layers (Fig. 7A). Following injury, H2AK119ub expression increased sharply at the leading edge (Fig. 7B), coinciding with DNA damage accumulation (Fig. 2B) and being consistent with a role in DNA repair [24, 25]. H2AK119ub remained elevated at PSD14 (Fig. 7C) but declined by PSD21 (Fig. 7D). By 2MPW, it was nearly absent in healed regions (Fig. 7E). H2AK119ub expression began to recover by 3MPW and partially returned by 6MPW, but remained below baseline (Fig. 7F, G, quantified in P). H2AK119ub dynamics indicate a PRC1-relieved, memory-like chromatin state with enhanced chromatin accessibility.

To further define the chromatin landscape, we examined histone H3 lysine 27 acetylation (H3K27ac), an active enhancer-associated modification [26, 27]. H3K27ac remained stable at PSD7 (Fig. 7H, I), declined markedly by PSD14 (Fig. 7J), and remained suppressed through 2MPW (Fig. 7K, L). Recovery began at 3-4MPW (Fig. 7M, N), and by 6MPW, H3K27ac approached baseline (Fig. 7O, quantified in P). We next examined histone H3 lysine 27 trimethylation (H3K27me3), the PRC2-catalyzed repressive mark opposing H3K27ac [28]. H3K27me3 showed modest increases at PSD4 and PSD14, peaking at 1-2MPW before returning to lower levels by 6MPW (Supplementary Fig. 8). This reciprocal relationship between H3K27ac and H3K27me3 suggests transient reinforcement of PRC2-mediated repression followed by release into a more permissive, memory-like chromatin state.

Together, these findings reveal persistent epigenetic remodeling during wound repair, indicating that delayed resolution of chromatin states contributes to prolonged molecular recovery after re-epithelialization.

## Discussion

**Fig. 8:**
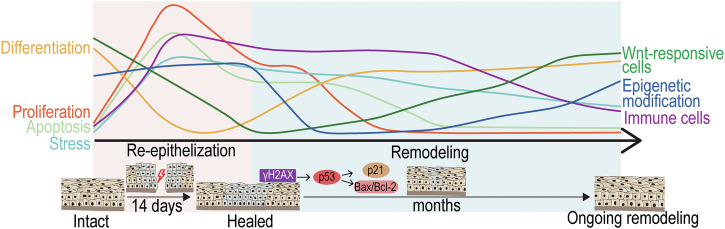
Proposed model of prolonged post-healing remodeling in oral mucosa. Schematic summary of events following wound closure. Although re-epithelialization is completed within two weeks, the regenerated epithelium remains in a repair-activated state with sustained apoptosis, stress-keratin expression, transient depletion of Wnt-responsive stem cells, and dynamic epigenetic remodeling. Gradual structural, cellular, and molecular remodeling ultimately restores epithelial homeostasis.

Through comprehensive lineage tracing, molecular profiling, and temporal analysis extending up to six months post-wounding, we systematically characterized the cellular and molecular dynamics of oral mucosal remodeling following wound closure (Fig. 8). While re-epithelialization completes within two weeks, full tissue homeostasis takes months, with certain molecular markers remaining altered even at six months. To our knowledge, this study provides the first integrated characterization of the prolonged remodeling phase after re-epithelialization, revealing coordinated elimination of repair cells, stem cell niche reconstruction, epigenetic reprogramming, and immune adaptation that together sustain regenerative homeostasis.

### Leading edge cell fate: evidence for programmed elimination

Leading edge cells represent a specialized population at the wound margin that drives wound re-epithelialization through coordinated migration and proliferation [11, 12, 29]. Our analysis shows that these cells acquire molecular features distinct from homeostatic epithelium: keratin 5 is expanded beyond the basal layer, while differentiation markers keratin 10 and filaggrin are lost, and stress keratins 6/17 are strongly induced (Fig. 5; Supplementary Fig. 4). These changes indicate a shift from a differentiated state to a repair-activated state.

Because wound closure occurs through the convergence of opposing epithelial fronts, it is not surprising that the newly healed epithelium initially inherits these altered signatures. Subsequently, our temporal analysis uncovered that these cells undergo selective elimination rather than integration into the restored epithelium. First, newly healed regions show transient hypercellularity followed by sustained apoptosis (Fig. 1), suggesting overproduction of repair cells and their subsequent removal. Second, lineage tracing reveals that Wnt-responsive cells are displaced from their niche and fail to integrate into the regenerated epithelium, with normal organization restored by 5MPW (Figs. 3–4). The prolonged absence of Wnt-responsive cells in healed tissue indicates that the post-healing microenvironment requires extensive remodeling before it can once again support functional stem cell niches. Third, the DNA damage-induced p53-p21 pathway remains robustly activated in newly healed tissue, mirroring its pattern in leading edge cells (Fig. 2). This sustained activation drives apoptotic elimination, consistent with previously reported mechanisms, in which p53 coordinates leader cell behavior and programmed clearance during epithelial repair [30].

The biological rationale for this elimination lies in the extensive molecular reprogramming and potential genomic instability acquired by leading edge cells during repair. Instead of restoring these transiently altered cells, the tissue replaces them with newly generated epithelial populations capable of reestablishing normal homeostasis.

### Tissue memory and persistent cellular remodeling in post-healed epithelium

Tissue memory—the retention of molecular and cellular imprints from prior injury—has emerged as a key regulator of epithelial plasticity and long-term homeostasis. Studies in skin have shown that stem cells acquire durable epigenetic and transcriptional changes that enhance subsequent repair responses [15, 16]. Our findings reveal a similar phenomenon in oral mucosa. The persistent reduction of H2AK119ub, together with H3K27ac and H3K27me3 dynamics, indicates that wound healing establishes a long-lasting “memory” state characterized by incomplete restoration of epigenetic regulation. The concurrent loss of repressive and active marks suggests that healed tissue remains in an intermediate chromatin state, with partial repair gene activation and subdued global transcription, reflecting ongoing remodeling rather than stable homeostasis.

This memory state persists until stem cell niches are fully reconstituted (Fig. 4). Wnt-responsive cells remain depleted for months after closure, and the remaining stem cells may adopt a heightened activation potential to compensate for their reduced numbers. Thus, epigenetic priming and altered chromatin accessibility could represent a compensatory mechanism maintaining tissue turnover and regenerative capacity despite stem cell scarcity.

### Implications for tumor formation: remodeling as a barrier against malignant transformation

In response to injury, diverse immune cell populations including macrophages and lymphocytes infiltrate the epithelium, promoting epithelial proliferation through secretion of various cytokines and growth factors, while also providing immune surveillance [31–34]. Our findings reveal that these immune cells persist within the healed tissue long after wound closure (Fig. 6). The prolonged retention of immune cells may represent an extended surveillance and regulatory mechanism that supports tissue remodeling and maintains epithelial integrity during homeostatic recovery. However, how these persistent immune populations interact with Wnt-responsive stem cells during long-term remodeling remains unclear and warrants further investigation.

In addition to immune persistence, the post-healing epithelium exhibits sustained stress-keratin expression and loss of differentiation markers, molecularly resembling the stress-activated [35–39], poorly differentiated [40, 41]. phenotype characteristic of head and neck squamous cell carcinoma. These parallels indicate that the healed tissue transiently adopts a tumor-like phenotype. However, unlike tumors, the healing epithelium undergoes an organized remodeling process that restores epithelial architecture, re-establishes stem-cell homeostasis, and resolves immune activation. This coordinated epithelial-immune remodeling reverses the transient pre-cancer-like state, preventing its progression toward malignancy. In contrast, cancer represents a pathological wound that fails to complete this resolution phase [42, 43]. remaining locked in a state of persistent stress, inflammation, and incomplete differentiation. Understanding these restorative processes may guide strategies to reprogram tumor microenvironments toward resolution by harnessing the tissue’s intrinsic capacity for remodeling.

### Limitations

Several limitations of this study should be noted. First, our lineage tracing approach, while informative, labeled only Axin2^+^ cells and may not capture the full spectrum of stem cells involved in wound repair. Including additional lineage markers such as Lgr5, Bmi1, or Lrig1 would provide a more comprehensive view of stem cell dynamics during remodeling. Second, although our analysis extends to six months post-injury, longer follow-up is needed to determine whether remodeling leads to full restoration or a new steady state with lasting changes. Finally, direct measurement of barrier permeability to assess full restoration of barrier function in healed tissue remains an important area for future investigation. Additionally, biomechanical properties and secondary injury responses of the remodeled tissue warrant further study to comprehensively assess functional restoration.

## Conclusion

This study revealed that oral wound healing involved two distinct phases: rapid re-epithelialization followed by prolonged cellular remodeling. The dynamic cellular and molecular changes we identified during this remodeling phase fundamentally reframe our understanding of tissue repair as an extended process rather than an event concluded at wound closure. These findings highlight the need to evaluate long-term tissue remodeling when assessing repair success and to develop therapeutic strategies aimed not only at achieving closure but also at ensuring complete restoration of tissue structure and function.

## Materials and methods

### Animals

All animal experimental protocols were approved by the Indiana University Institutional Animal Care and Use Committee (#24012). Both male and female C57BL/6 J wild-type mice and *Axin2*^*CreERT2*^*;EGFP* lineage tracing mice [22]. on a C57BL/6 J background were used in this study.

### Lineage tracing

Tamoxifen (T5648, Sigma-Aldrich, St. Louis, MO, USA) was dissolved in 100% ethanol and then diluted with sunflower seed oil to a final concentration of 10 mg/ml. A single intraperitoneal dose of 5 mg tamoxifen per 25 g body weight was administered. Our previous studies demonstrated that this regimen efficiently induced Cre-mediated recombination [7, 22]. The pulse-chase timelines are illustrated in figures and legends.

### Surgeries

Mice aged between 8-12 weeks were anesthetized with ketamine (100 mg/kg) and xylazine (10 mg/kg), followed by pre-operative analgesia with buprenorphine SR (1 mg/kg, subcutaneously) and carprofen (10 mg/kg, subcutaneously). The oral cavity was opened using a magnetic retraction system (Fine Science Tools, Foster City, CA, USA). A 2 mm full-thickness excisional wound was created on the hard palate using a sterile biopsy punch (Integra LifeSciences, Princeton, NJ, USA). The injury site was localized at the intersection of the midline and the midpoint between the bilateral first molars (M1), corresponding anatomically to the region between rugae 4-7. The position was determined using both the molar teeth and the rugae pattern as reliable anatomical landmarks to ensure reproducibility and accuracy of wound placement (Supplementary Fig. 1A). Hemostasis was achieved by gentle pressure. Post-operatively, mice received an additional dose of carprofen 24 h after surgery.

### Sample preparation

Specimens were decalcified in 0.5 M EDTA (pH 7.2) for 3–5 days, followed by dehydration in a graded ethanol series, clearing in xylene, and infiltration with a xylene-paraffin mixture. Samples were subsequently embedded in paraffin, and sagittal sections (6 µm thick) were prepared and mounted onto positively charged glass slides.

### Hematoxylin and eosin (H&E) stain

Paraffin sections were deparaffinized in xylene and rehydrated through descending concentrations of ethanol. Slides were immersed in hematoxylin solution for 4 minutes, rinsed with running tap water, and differentiated in 1% acid alcohol. After an additional rinse, sections were transferred to 95% ethanol and counterstained with eosin Y for 10 seconds. Sections were then dehydrated, cleared in xylene, and mounted with Permount mounting medium.

### Masson’s trichrome staining

Paraffin-embedded tissue sections were first deparaffinized and rehydrated through a series of graded ethanol solutions. Masson’s Trichrome staining was then performed using the Masson’s Trichrome for Connective Tissue Kit (Product #26367, Electron Microscopy Sciences, Hatfield, PA, USA) following the manufacturer’s instructions. Following staining, sections were dehydrated, cleared with xylene, and coverslipped using a Permount mounting medium.

### EdU labeling and detection

Cell proliferation was assessed using a 5-ethynyl-2’-deoxyuridine (EdU) incorporation assay, which labels replicating DNA [44]. Mice received an intraperitoneal injection of EdU (50 mg/kg in PBS). Tissues were collected half an hour later. Samples were fixed and processed as described above. Sections were permeabilized with 0.5% Triton X-100 and incubated for 30 minutes with a Click-iT reaction cocktail prepared from the EdU in vivo kit (BCK-IV-IM, Base Click, Neuried, Germany) according to the manufacturer’s instructions. Slides were subsequently washed and mounted with ProLong™ Gold antifade mountant containing DAPI (Invitrogen, Carlsbad, CA, USA).

### Cell apoptosis

Apoptotic cells were identified using terminal deoxynucleotidyl transferase dUTP nick end labeling (TUNEL) with the In Situ Cell Death Detection Kit (11684795910, Roche, Indianapolis, IN, USA). Briefly, sections were permeabilized using a freshly prepared Triton X-100/sodium citrate solution (0.1% Triton X-100, 0.1% sodium citrate) for 8 min at room temperature, followed by incubation with the TUNEL reaction mixture at 37 °C for 1 h in the dark. For co-staining experiments, immunofluorescence was performed first, followed by the TUNEL reaction. After washing, slides were mounted with ProLong™ Gold antifade mountant containing DAPI.

### Immunohistochemistry and immunofluorescence staining

Paraffin-embedded tissue sections were deparaffinized in xylene and rehydrated through graded ethanol to distilled water, followed by permeabilization with Triton X-100. Antigen retrieval was performed using an antigen unmasking solution (pH 6.0, H-3300, Vector Laboratories, Newark, CA, USA) in a water bath maintained at sub-boiling temperature for 15 min. Sections were then allowed to cool to room temperature and washed with PBS. Tissue sections were blocked with 5% goat serum (Jackson ImmunoResearch, 005-000-121) at room temperature for half an hour and then incubated with primary antibodies at 4 °C overnight. On the following day, sections were washed and incubated with secondary antibodies at room temperature. The following primary antibodies were utilized: anti-E-cadherin (1:100, #3195, Cell Signaling Technology, Danvers, MA, USA), anti-filaggrin (1:600, 905804, BioLegend, San Diego, CA, USA), anti-keratin 10 (1:400, 905404, BioLegend), anti-keratin 6 (1:300, MA5-16373, Invitrogen), keratin 17 (1:100, #4543, Cell Signaling Technology), anti-keratin 5 (1:800, 905504, BioLegend), anti-Laminin 5 (1:100, ab14509, Abcam, Waltham, MA, USA), anti-GFP (1:100, #2956, Cell Signaling Technology), anti-H3K27me3 (1:50, A22006, Abclonal, Woburn, MA, USA), anti-H2AK119ub (1:700, #8240, Cell Signaling Technology), anti-H3K27ac (1:300, A2771, Abclonal), anti-p53 (1:100, GTX638291, GeneTex), anti-p21 (1:50, 188224, Abcam), anti-Bcl2 (1:200, ab182858, Abcam), anti-Bax (1:200, A19684, Abclonal), anti-active caspase 3 (1:50, GTX03281, GeneTex), anti-Cyclin D1 (1:300, A11022, Abclonal), anti-γH2AX (1:500, AP0687, Abclonal), anti-CD45 (1:100, #70257, Cell Signaling Technology), anti-CD3 (1:200, #78588, Cell Signaling Technology), anti-F4/80 (1:400, #70076S, Cell Signaling Technology), anti-MPO (1:200, A22900, Abclonal), anti-CD20 (1:500, #70168, Cell Signaling Technology), anti-Type I collagen (1:400, #72026, Cell Signaling Technology), anti-Vimentin (1:100, #5741, Cell Signaling Technology), and anti-PCNA (1:400, PA-27214, Invitrogen). For visualization, species-specific secondary antibodies were applied according to the detection method. For fluorescence immunostaining, Alexa Fluor Plus 647-conjugated goat anti-rabbit IgG (H + L), highly cross-adsorbed (A32733, Invitrogen), was used. For chromogenic detection with DAB, sections were incubated with HRP-conjugated goat anti-rabbit IgG (H + L) (111-035-144, Jackson ImmunoResearch, West Grove, PA, USA), followed by color development using the ImmPACT DAB Substrate (SK-4105, Vector Laboratories).

### Quantitative real-time PCR

For gene expression analysis, hard and soft palates were carefully dissected from the palatal bone and incubated with 2.4 IU dispase II (Roche, Indianapolis, IN) at 37 °C for 30 minutes with shaking to separate the epithelial layer from the underlying connective tissue [7]. The epithelial layer from healed regions was isolated using a 2 mm biopsy punch to match the original wound diameter. Epithelial samples were immediately placed in steel bead tubes (Benchmark Scientific, D1033-28) containing 400 μl RNA Lysis Buffer from the Quick-RNA Miniprep Kit (Zymo Research, Irvine, CA) and homogenized using a BeadBug microtube homogenizer (Benchmark Scientific, D1030) at 4000 rpm for three 30-second cycles with 30-second intervals on ice.

Total RNA was extracted following the manufacturer’s protocol with on-column DNase I digestion to eliminate genomic DNA contamination. RNA concentration and purity were assessed using a SmartDrop spectrophotometer (LabX, Midland, ON, Canada), with A260/280 ratios between 1.8–2.0 considered acceptable. First-strand cDNA synthesis was performed using 500 ng total RNA with the LunaScript RT SuperMix Kit (New England Biolabs, E3010G, Ipswich, MA) according to the manufacturer’s instructions.

Quantitative PCR was performed using the Luna Universal qPCR Master Mix (New England Biolabs, M3003) on a QuantStudio 3 Real-Time PCR System (Thermo Fisher Scientific, Waltham, MA). Five biological samples per group were analyzed, and reactions were carried out in triplicate. Cycling conditions were: 95 °C for 1 minute, followed by 40 cycles of 95 °C for 15 seconds and 60 °C for 30 seconds. Melt curve analysis was performed to verify amplicon specificity. Gene expression levels were normalized to *Gapdh* as the housekeeping gene and calculated using the 2^-ΔΔCt^ method. Data are presented as fold change relative to intact tissue.

### Quantification

To assess basal cell density and epithelial thickness, representative images from the wound center were imported into Fiji (ImageJ, NIH, Bethesda, MD, USA) with scale bars for accurate measurements. Basal cell density was quantified as the number of basal keratinocytes per 1 mm length of basement membrane. Epithelial thickness was measured as the distance from the basement membrane to the stratum corneum at three random locations per field. Quantification was performed on two slides per mouse, with measurements averaged per animal, using a total of 8 mice per experimental group.

Quantification of EdU^+^ and TUNEL^+^ cells, EdU^+^ or TUNEL^+^ cells and DAPI^+^ epithelial cells were counted using Fiji, with positive cells expressed as a ratio to total DAPI+ epithelial cells. Two slides per mouse were assessed, with a total of 8 mice per group. For PSD4 and PSD7, 500 µm of tissues from leading edge were examined. Quantification of CD45, CD3, and F4/80 positive cells was performed using Fiji, with cell counts normalized to basal membrane length. Quantification of H2AK119ub and H3K27ac positive cells were performed with Fiji, with positive cells expressed as a ratio to total DAPI^+^ epithelial cells. Two slides per mouse were assessed, with a total of 8 mice per group.

To examine Wnt-responsive cells and their progeny, EGFP^+^ cells were counted using Fiji. EGFP^+^ basal cells were defined as EGFP^+^ cells associated with the basal membrane, with this number normalized to the total number of basal cells. EGFP^+^ clones were defined as clusters containing at least one EGFP^+^ cell associated with the basal membrane. EGFP^+^ clone size was quantified by counting the number of EGFP^+^ cells within each clone. Three slides per mouse were assessed, with a total of 6 mice per group.

For quantification of keratin 5, 10, and 6, positive pixels were measured using Fiji and normalized to the total pixels of the epithelial tissue using the method we reported previously [45]. Two slides per mouse were assessed, with a total of 8 mice per group.

All image acquisition and quantitative analyses were performed in a blinded manner, with samples coded prior to analysis to conceal group allocation.

### Statistical analysis

All data are presented as means $\pm$ standard deviation (SD). Sample size was determined based on prior experience with this experimental model and published studies using similar designs. No samples were excluded from the analysis. Mice were randomly assigned to experimental time points using a computer-generated random number generator. Allocation was performed prior to surgery. Data distribution was assessed for normality prior to parametric testing. Variance was comparable between groups. For comparisons between two groups, two-tailed unpaired Student’s *t*-test was used. For comparisons involving more than two groups, one-way analysis of variance (ANOVA) followed by Tukey’s multiple comparisons test was performed to adjust for multiple testing. Statistical analyses were conducted using Prism 10 (GraphPad Software, La Jolla, CA, USA). Exact *P* values are shown in the figures. A *P* value < 0.05 was considered statistically significant.

## Supplementary information

Supplemental material

## Data Availability

All data needed to evaluate the conclusions in the paper are present in the paper and the supplementary materials.
