# Supplementary material for: Beyond re-epithelialization: prolonged remodeling re-establishes epithelial homeostasis in oral mucosa
Source: Cell Death Dis. 2026 Apr 28;17(1):569. doi: 10.1038/s41419-026-08804-z (PMC13265752; doi:10.1038/s41419-026-08804-z)
Supplement: Supplementary file 1 — Supplemental material [file 41419_2026_8804_MOESM1_ESM.pdf]

SUPPLEMENTARY FIGURES AND FIGURE LEGENDS

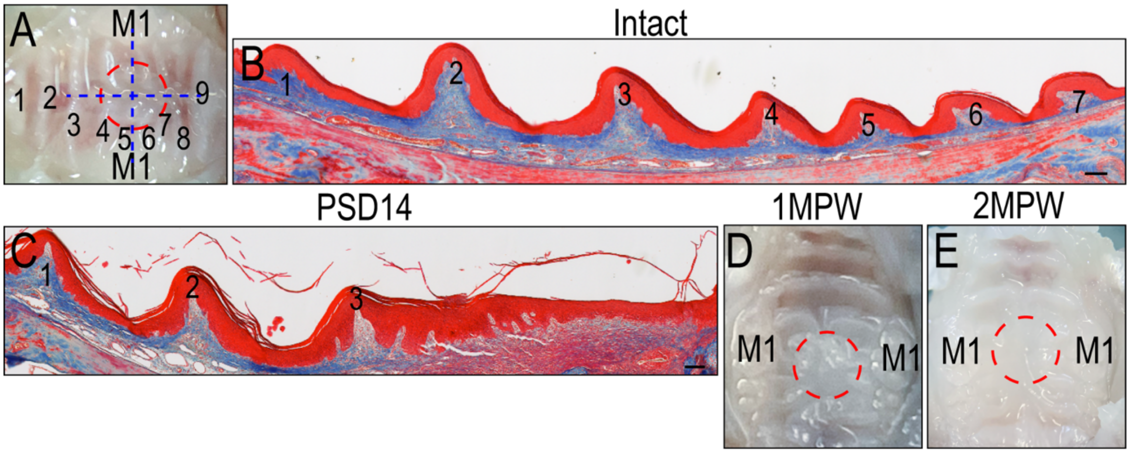

Supplementary Figure 1. Establishment and characterization of the mouse hard palate wound healing model. (A) Illustration of the intact hard palate showing molars and the rugae pattern, which serve as anatomical landmarks for precise localization of the injury site (dashed red circle). (B) Masson's trichrome-stained section of the intact palate showing normal rugae morphology (numbers 1-7). (C) Representative Masson's trichrome-stained section of the palate at post-surgery day 14 (PSD14). (D, E) Gross images of the hard palate at 1 month post-wound (1MPW) and 2MPW showing the healed wound area (dashed red circle). M1, first molar. Numbers 1-9 indicate hard palate rugae 1-9, respectively. Scale bars, 50 μm.

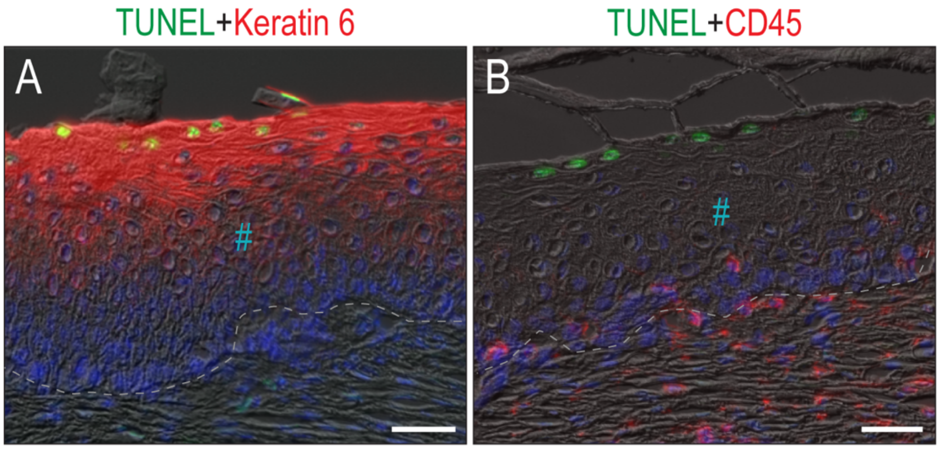

Supplementary Figure 2. Lineage identification of apoptotic cells. Representative immunofluorescence staining of healed region at PSD14 showing TUNEL (green) co-stained with (A) keratin 6 (red) and (B) CD45 (red). Nuclei are counterstained with DAPI (blue). The healed area is marked by # symbols. Scale bars, 25 μm.

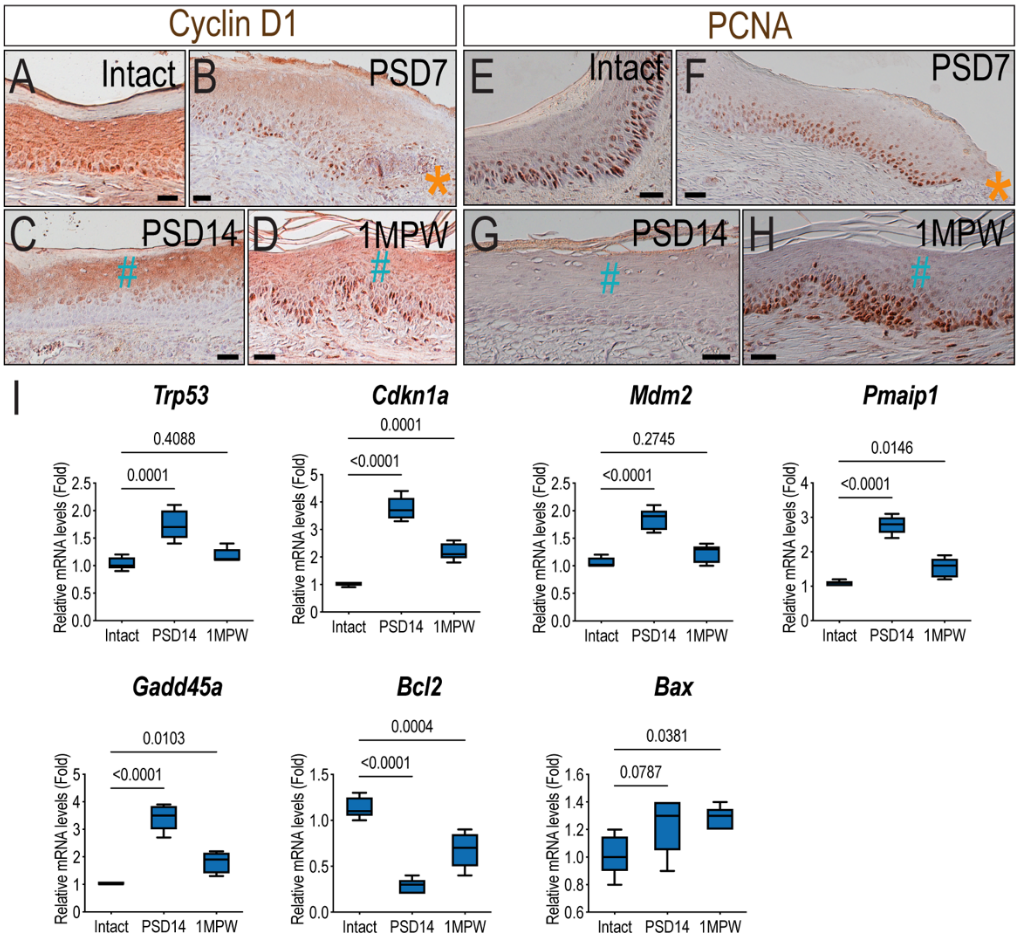

Supplementary Figure 3. Epithelial proliferation marker dynamics during re-epithelialization and remodeling. (A-D) Representative immunohistochemical images showing Cyclin D1 expression in intact epithelium and at post-surgery days (PSD) 7 and 14, and 1 month post-wound (1MPW). (E-H) Representative immunohistochemical images showing PCNA expression at corresponding time points. (I) Quantitative PCR analysis of cell cycle regulators and apoptosis markers at Intact, PSD14, and 1MPW. Expression levels of *Trp53*, p53 target genes (*Cdkn1a*, *Mdm2*, *Pmaip1*, *Gadd45a*), and apoptosis-related genes (*Bcl2*, *Bax*) were normalized to *Gapdh*. Data are presented as fold change relative to intact tissue (n = 5). Asterisks (*) denote original wound sites. Pound signs (#) indicate healed epithelial regions. Scale bars, 50 μm.

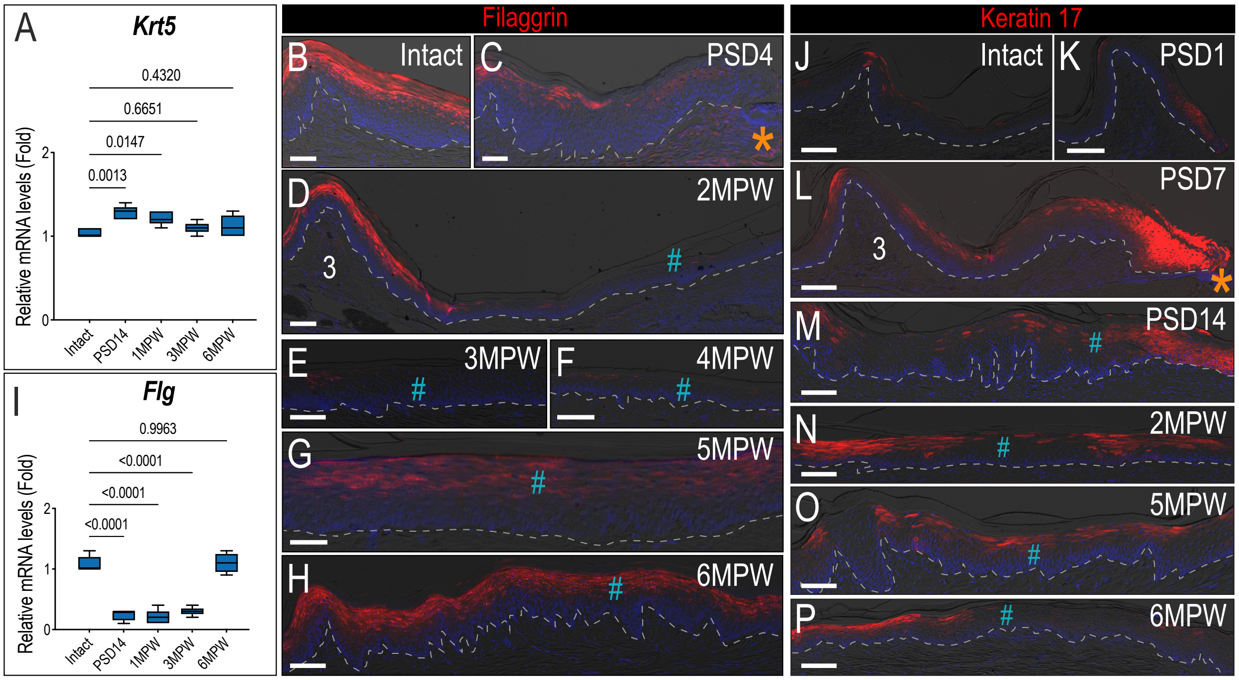

Supplementary Figure 4. Epithelial marker dynamics during re-epithelialization and remodeling. (A) Quantitative PCR analysis of keratin 5 (*Krt5*) expression at intact, PSD14, 1MPW, 3MPW, and 6MPW time points. Expression levels normalized to *Gapdh*. Data are presented as fold change relative to intact tissue (n=5). (B-H) Representative immunofluorescence staining of filaggrin at the indicated time points. (I) Quantitative PCR analysis of filaggrin (*Flg*) expression at intact, PSD4, 1MPW, 3MPW, and 6MPW. Expression levels were normalized to *Gapdh*. Data shown as fold change relative to intact tissue (n=5). (J-P) Representative immunofluorescence staining of keratin 17 (red) in intact epithelium and at indicated time points. Dashed lines outline the epithelial-stromal boundary. The healed area is marked by # symbols. Asterisks (*) denote wound sites. Scale bars, 50 μm.

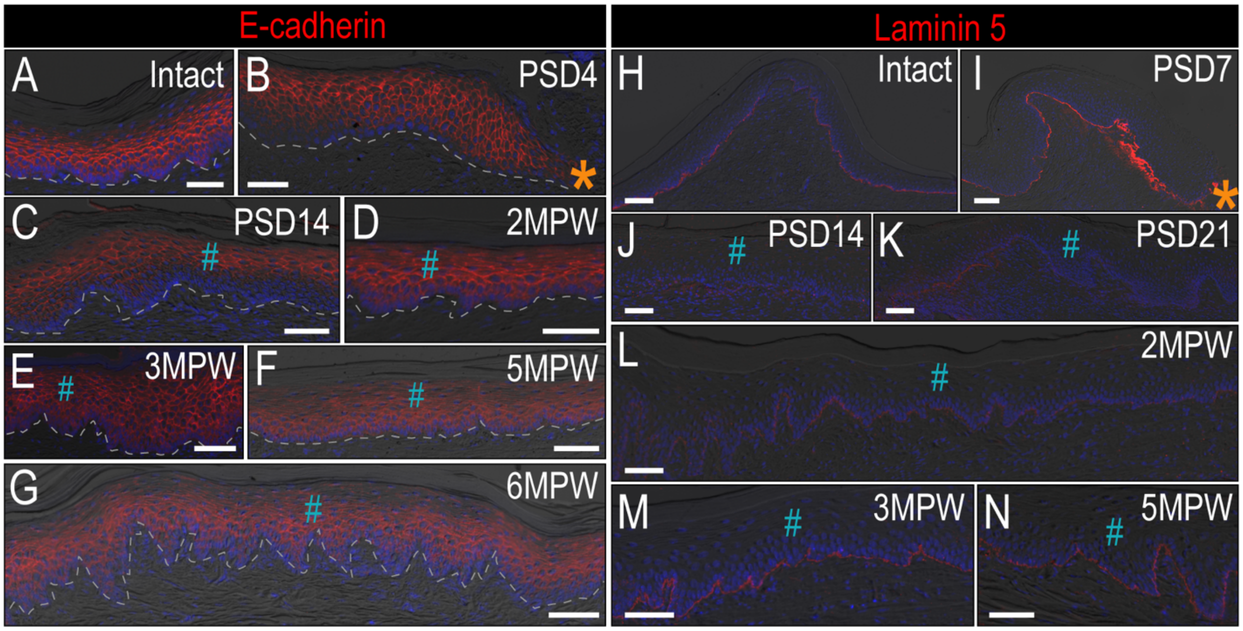

Supplementary Figure 5. Dynamics of epithelial protein expression during re-epithelialization and remodeling. Representative immunofluorescence staining of (A-G) E-cadherin and (H-N) Laminin 5 at the indicated time points. The healed area is marked by # symbols. Asterisks (*) denote wound sites. Scale bars, 50 μm.

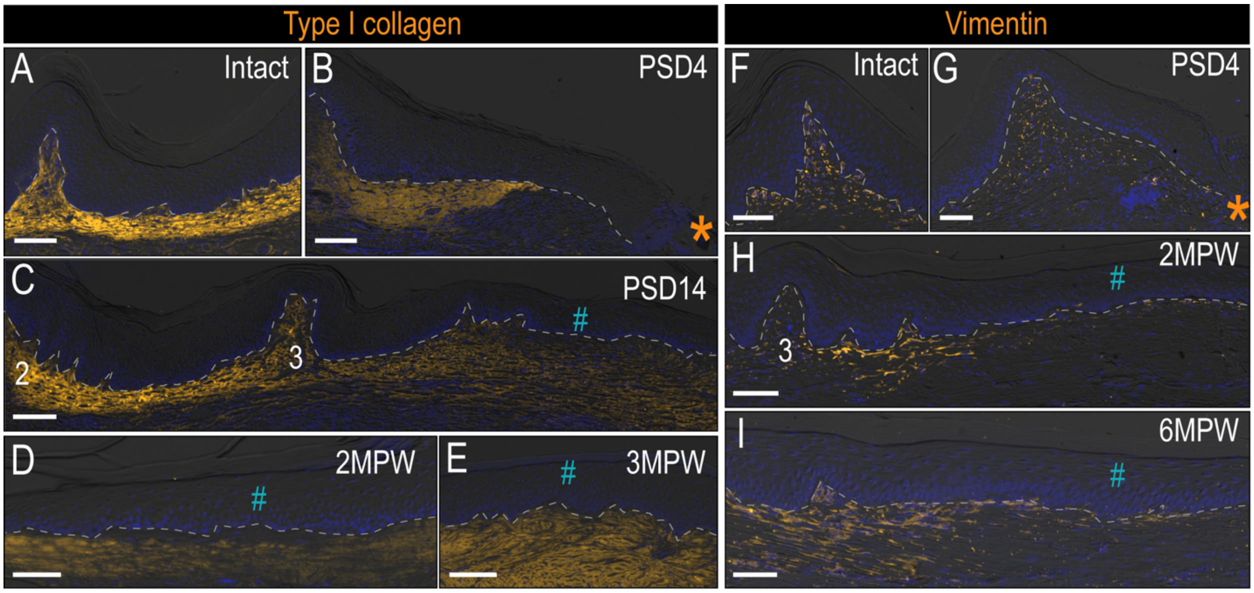

Supplementary Figure 6. Connective tissue dynamics during re-epithelialization and remodeling. Representative immunofluorescence staining of (A-E) Type I collagen in the connective tissue and (F-I) Vimentin showing mesenchymal cells at the indicated time points. The healed area is marked by # symbols. Asterisks (*) denote wound sites. Scale bars, 50 μm.

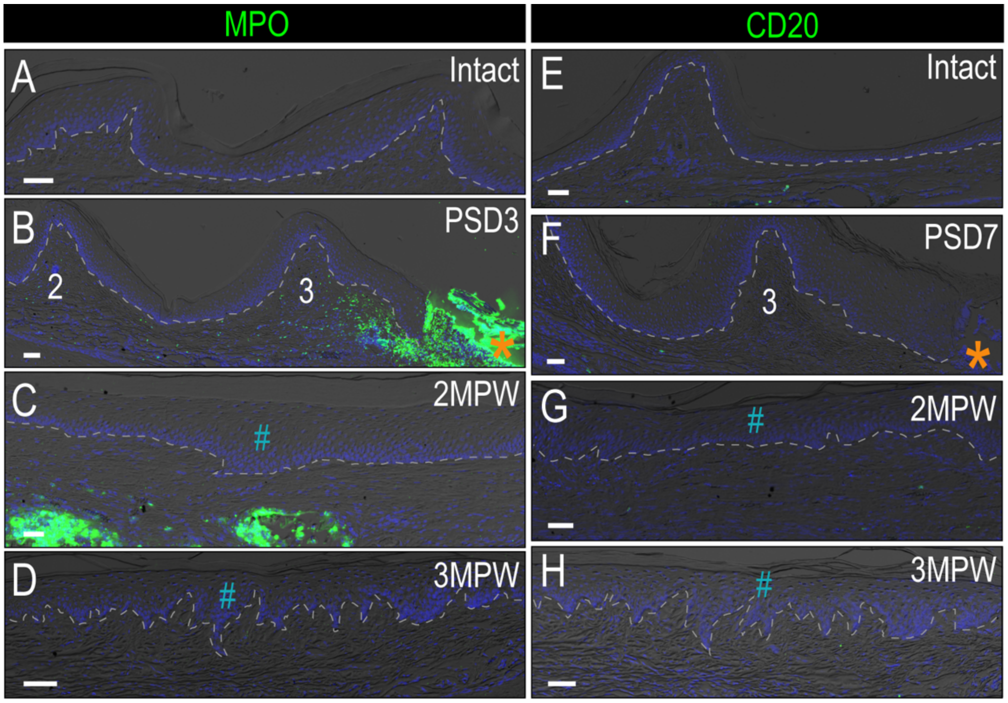

Supplementary Figure 7. Neutrophil and B cell infiltration during re-epithelialization and remodeling. Representative immunofluorescence staining of (A-D) myeloperoxidase (MPO) for neutrophils and (E-H) $CD20^{+}$ B cells at the indicated time points. Dashed lines outline the epithelial–stromal boundary. The healed area is marked by # symbols. Numbers 2 and 3 indicate hard palate rugae landmarks. Asterisks (*) denote wound sites. Scale bars, 50 μm.

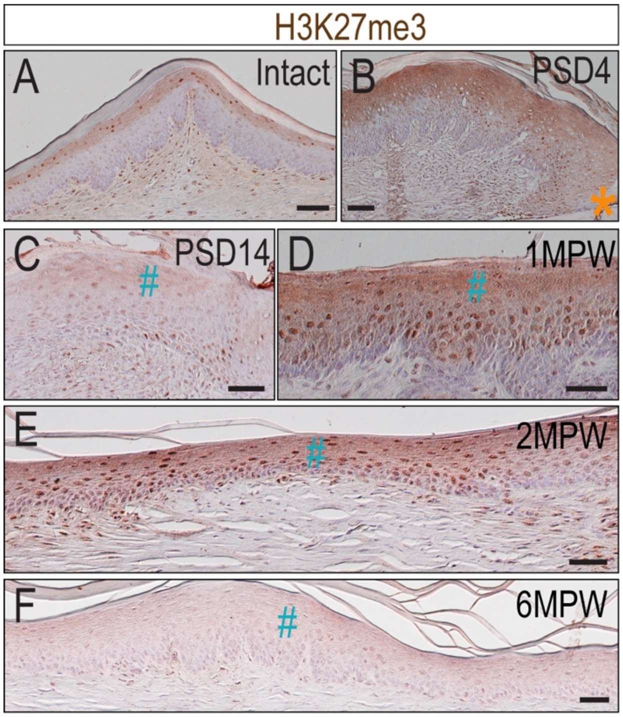

Supplementary Figure 8. Temporal dynamics of H3K27me3 expression during re-epithelialization and remodeling. (A-F) Representative immunohistochemical staining of H3K27me3 in the oral epithelium at the indicated time points. The healed area is indicated by # symbols, and asterisks (*) denote original wound sites. Scale bars, 50 μm.
